# Pharmacogenomics in colorectal cancer: current role in clinical practice and future perspectives

**DOI:** 10.20517/2394-4722.2018.04

**Published:** 2018-03-07

**Authors:** Francesca Battaglin, Alberto Puccini, Madiha Naseem, Marta Schirripa, Martin D. Berger, Ryuma Tokunaga, Michelle McSkane, Taline Khoukaz, Shivani Soni, Wu Zhang, Heinz-Josef Lenz

**Affiliations:** 1 Division of Medical Oncology, Norris Comprehensive Cancer Center, Keck School of Medicine, University of Southern California, Los Angeles, CA 90033, USA; 2 Medical Oncology Unit 1, Clinical and Experimental Oncology Department, Veneto Institute of Oncology IOV - IRCCS, Padua 35128, Italy; 3 Department of Medical Oncology, University Hospital of Bern, Bern 3010, Switzerland

**Keywords:** Colorectal cancer, pharmacogenomics, RAS, BRAF, microsatellite instability, dihydropyrimidine dehydrogenase, UDP-Glucuronosyltrasferase A1, epidermal growth factor receptor, vascular endothelial growth factor, DNA methylation

## Abstract

The treatment scenario of colorectal cancer (CRC) has been evolving in recent years with the introduction of novel targeted agents and new therapeutic strategies for the metastatic disease. An extensive effort has been directed to the identification of predictive biomarkers to aid patients selection and guide therapeutic choices. Pharmacogenomics represents an irreplaceable tool to individualize patients treatment based on germline and tumor acquired somatic genetic variations able to predict drugs response and risk of toxicities. The growing knowledge of CRC molecular characteristics and complex genomic makeup has played a crucial role in identifying predictive pharmacogenomic biomarkers, while supporting the rationale for the development of new drugs and treatment combinations. Clinical validation of promising biomarkers, however, is often an issue. More recently, a deeper understanding of resistance mechanisms and tumor escape dynamics under treatment pressure and the availability of novel technologies are opening new perspectives in this field. This review aims to present an overview of current pharmacogenomic biomarkers and future perspectives of pharmacogenomics in CRC, in an evolving scenario moving from a single drug-gene interactions approach to a more comprehensive genome-wide approach, comprising genomics and epigenetics.

## INTRODUCTION

Colorectal cancer (CRC) is one of the leading causes of cancer-related deaths in the western world and ranks third among the most frequent malignancies in both men and women^[1]^. Although still unsatisfactory, the median overall survival (OS) of patients with metastatic CRC (mCRC) has notably increased in the past 20 years, reaching around 30 months in recent phase III clinical trials^[2,3]^, thanks to the introduction of innovative medical and surgical treatment strategies. The availability of new drugs and treatment combinations, both in terms of cytotoxic chemotherapy regimens and new targeted therapies, has been crucial in order to reach this result. However, patients’ outcome and response to treatment can be highly heterogeneous, thus an extensive effort has been directed towards the identification of reliable predictive biomarkers to aid clinical management of patients and identify subgroups more likely to benefit from different treatment strategies.

Pharmacogenomics represents an irreplaceable tool in order to tailor patients treatment to an individualized approach based on germline and somatic acquired genetic variations able to predict drugs response and risk of toxicities^[4]^. Moving from early studies exploring the genetic bases of individual predisposition to severe toxicities from chemotherapy agents [i.e. 5-fluorouracil (5-FU) or irinotecan] in mCRC patients, the introduction of targeted agents such as anti-epidermal growth factor receptor (EGFR) drugs, has prompted the discovery of predictive molecular biomarkers (i.e. RAS mutational status) which are now tested as part of routine clinical practice^[5]^. Over time, additional mechanisms of primary and secondary resistance to targeted agents have emerged as promising novel predictive biomarkers and potentially actionable target of treatment, although validation is still an issue in most cases, and many steps forward have been made in the biological understanding and molecular characterization of CRC^[6]^. Finally, new perspectives have been recently opened following innovative results of immunotherapy treatment, and the development of new analytical techniques which allow dynamic tumor profiling and a sensitive detection of coexisting alterations underlying tumor heterogeneity, such as liquid biopsy^[7]^.

In this review, we present an overview of current pharmacogenomic biomarkers validated in clinical practice and future perspectives of pharmacogenomics in CRC [Tables 1 and 2], in an evolving scenario moving from a single drug-gene interactions approach to a more comprehensive genome-wide approach, comprising genomics and epigenetics.

## CURRENT PHARMACOGENOMIC BIOMARKERS IN CLINICAL PRACTICE RAS

EGFR signaling pathway plays a crucial role in the regulation of cellular responses to growth signals and its constitutive activation is one of the main actor promoting CRC growth and proliferation through the KRAS/RAF/MAPK and the PI3K/AKT/mTOR axes^[8]^. EGFR inhibitors are nowadays well-established therapeutic agents incorporated into standard care for mCRC^[9,10]^. To date, two anti-EGFR monoclonal antibodies have been approved by the Food and Drug Administration (FDA) and European Medicines Agency (EMA) for the treatment of mCRC: Cetuximab (Erbitux®, Merck KGaA/Lilly USA) and Panitumumab (Vectibix®, Amgen Inc). At the time when the efficacy of these drugs was first proven in advanced lines of treatment^[11,12]^, no predictive biomarker was available, although a subgroup effect on the activity of these agents was evident. KRAS is a small GTPase member of the RAS protein family^[13]^, and somatic gene mutations can lead to its constitutive activation resulting in independent cell proliferation and survival^[14]^. *KRAS* mutations, more frequently involving exon 2^[15]^, can be found in approximately 40% to 50% of mCRCs. The identification of *KRAS* exon 2 (codons 12 and 13) mutations as a negative predictive marker of response to anti-EGFRs represented the turning point on biomarker selection for anti-EGFR treatment.

First evidence of the negative predictive role of *KRAS* exon 2 mutation came from retrospective series^[16]^ and was then confirmed through *post-hoc* analyses of randomized phase III trials^[11,17–20]^. Moving from these data, in 2008 FDA and EMA restricted the use of anti-EGFR drugs to patients with *KRAS* exon 2 wild-type (WT) tumors. However, in the same year, the possible existence of additional predictive biomarkers of resistance to anti-EGFR treatment was highlighted by an independent meta-analysis^[21]^ showing a low sensitivity for *KRAS* exon 2 mutations in predicting acquired resistance to anti-EGFRs. Shortly after, rare *RAS* activating mutations in exon 3 (codons 59 and 61) and exon 4 (codons 117 and 146) of *KRAS* and exons 2, 3, and 4 of *NRAS* (codons 117 and 146), were reported as novel negative predictive markers^[22,23]^. Outcome data from the extended *RAS* analyses in the large randomized phase III PRIME trial, comparing FOLFOX with or without panitumumab as first-line treatment in mCRC patients, provided definitive evidence in this regard. In this study, patients with any *RAS* mutation in their tumors showed a worse outcome when treated with panitumumab [hazard ratio (HR) for progression free survival (PFS) = 1.31 (*P* = 0.008, *P* for interaction < 0.002); HR for OS = 1.21 (*P* = 0.04, *P* for interaction = 0.001)]^[24]^. Following this evidence, results of all recent randomized trials with anti-EGFR-based therapies were retrospectively re-evaluated according to the extended *RAS* mutational status^[25–27]^ and several meta-analyses were performed. Data were consistent across different chemotherapy backbones, anti-EGFR agents and lines of therapy, showing no improvement in outcome results, both in term of PFS and OS, with the addition of anti-EGFRs in tumors harboring any *RAS* mutation (*P* > 0.05)^[28]^. Notably, in the selected extended *RAS* WT population efficacy results from the addition of anti-EGFR treatment were highly improved^[29]^. Based on these results, the use of anti-EGFRs has been currently restricted to *RAS* WT (exons 2, 3, and 4 of each *KRAS* and *NRAS*) tumors^[30]^, and regulatory authorities recommend that every patient being considered for anti-EGFR therapy must receive *RAS* mutational testing including *KRAS* and *NRAS* codons 12, 13 of exon 2; 59, 61 of exon 3; and 117 and 146 of exon 4, performed only in highly qualified and certified laboratories^[5]^.

More recently, KRAS mutations have been shown to be associated with suppressed Th1/cytotoxic immunity in CRC, irrespective of mismatch repair (MMR) status, tumor location, neoantigen load and transcriptional subtype, with a differential effect modulated by the underlying tumor consensus molecular subtypes (CMS, discussed more extensively in section 4)^[31]^. These findings may have a role in explaining the heterogeneity of treatment response and outcomes in *RAS* mutated tumors and provide a rationale for novel treatment strategies in these patients.

## BRAF

The serine/threonine protein kinase BRAF is another player in the EGFR-mediated signaling pathway which is well-known to be implicated as an oncogenic driver in CRC. In normal cells, MEK, ERK and RAF are part of a tyrosine kinase signaling cascade activated by RAS, which affects cell proliferation, growth and differentiation, and regulates key cellular function such as apoptosis, cell migration and survival^[32]^. Mutations in *BRAF* can be found in approximately 8%−10% of CRCs^[33]^, the majority of which (about 80%) involve the substitution of glutamic acid for valine at residue 600 within the protein kinase domain (V600E). BRAF constitutive activation resulting from V600E mutation promotes signaling transduction through the MEK-ERK-MAP kinase pathway even in absence of RAS-mediated signals. RAS and BRAF V600E mutations, as they work through the same pathway, are considered mutually exclusive, and their concomitant detection is extremely rare (< 0.001%)^[34]^.

The negative prognostic value of *BRAF* V600E mutation in mCRC has been extensively described in several univariate and multivariate models. Life expectancy for this subgroup of patients is poor when compared to *BRAF* WT ones. When retrospectively evaluated, in fact, metastatic *BRAF*-mutated patients were showed to have a median OS ranging from 10 to 19 months across multiple series, even when treated with association therapies^[35–38]^. Additionally, *BRAF* V600E-mutated tumors share distinct clinicopathological features: they are more frequent in women, elderly, and are often right-sided; they more often present a mucinous histology, poor differentiation and high microsatellite instability (MSI-H); more often are diagnosed as advanced disease with preferential spread to lymph nodes and peritoneum^[39–41]^. When oligo-metastatic liver disease is radically resected, *BRAF*-mutated tumors tends to relapse early with extra-hepatic lesions^[42,43]^. A specific carcinogenesis pathway^[44]^ and a distinct gene signature^[45]^ have also been associated with *BRAF* V600E mutation. More recently, gene expression analyses allowed to identify two different *BRAF* V600E subtypes in a large cohort of *BRAF* V600E mutated patients unselected for tumor stage: the BM1 subtype characterized by KRAS/AKT activation, mTOR/4EBP deregulation and EMT, and the BM2 subtype characterized by cell cycle and checkpoint pathway deregulation^[46]^. In contrast with *BRAF* V600E mutation, metastatic tumors harboring rare mutations of *BRAF* codons 594 and 596 (less than 1% of CRCs) have been shown to have different prognosis and clinical outcome. These rare mutations are associated with a nonmucinous histology, a rectal primary tumor location, microsatellite stability, and lack of peritoneal disease. Moreover, no negative prognostic impact was observed although in a small series of patients (median OS 62.0 *vs.* 12.6 months; HR, 0.36; 95% CI, 0.20–0.64; *P* = 0.002 for *BRAF* 594 or 596 mutant *vs. BRAF* V600E)^[47]^. Similar results on the impact and characteristics of *BRAF* nonV600E mutations were confirmed in a recent retrospective evaluation of a large cohort of patients^[48]^.

Although still debated, growing evidence is accumulating on the role of *BRAF* mutations as a negative predictive marker for anti-EGFR agents activity. Retrospective series showed that the response rate to anti-EGFR treatment with or without chemotherapy was significantly lower in *BRAF*-mutated *vs.* WT patients^[22,23,49]^. On the other hand, *BRAF* V600E mutation failed to demonstrate its predictive value in several sub-group analyses of phase III trials, possibly because of the small number of *BRAF*-mutated patients and lack of statistical power^[24,50]^. More recently, two meta-analyses showed a lack of improvement in PFS and OS in patients with *BRAF*-mutated mCRCs when treated with either cetuximab- or panitumumab-containing regimens compared to chemotherapy alone^[51,52]^. Additionally, a retrospective evaluation of the randomized phase III FIRE-3 trial, comparing FOLFIRI plus cetuximab or bevacizumab as first-line treatment in KRAS exon 2 WT mCRC patients, confirmed poorer survival outcomes for *BRAF*-mutated tumors irrespective of cetuximab and bevacizumab administration^[53]^. Based on these data, it appears that anti-EGFRs do not demonstrate a clear outcome benefit in *BRAF*-mutated tumors, and their use should be restricted to patients with no alternative therapeutic options. Notably, however, in FIRE-3 cetuximab arm a small subgroup of *BRAF*-mutated tumors achieving an early tumor shrinkage ≥ 20% (9/17) showed significantly longer median PFS (9.0 *vs.* 1.9 months, log-rank test *P* = 0.002; HR = 0.14) and OS (29.8 *vs.* 5.9 months, log-rank test *P* = 0.047; HR = 0.3) than those not achieving it^[53]^. Despite the limitations due to the retrospective nature of this evaluation and the small patients numbers, these results highlight a significant heterogeneity among *BRAF*-mutated mCRCs warranting further investigation.

While FOLFOXIRI plus bevacizumab represents the most promising treatment option in the first-line setting for clinically selected *BRAF*-mutated patients^[2,54]^, outcomes are still unsatisfactory. An extensive effort has been made in the last few years aiming to develop possible effective anti-*BRAF* strategies for mCRC patients. In contrast to melanoma, the use of *BRAF* inhibitors, such as vemurafenib and dabrafenib, as single-agents did not show significant activity in *BRAF*-mutated mCRC^[55]^. Dual blockade of *BRAF* and alternative survival pathways, such as MEK and EGFR, have been tested as well in clinical trials without convincing results^[56–58]^. Promising results are coming instead from a triple inhibition strategy combining *BRAF*-inhibitors, MEK-inhibitors and EGFR-inhibitors^[59,60]^. An additional strategy under study to increase the activity of dual targeted *BRAF* inhibition is its association with standard cytotoxic chemotherapy, such as the combination of vemurafenib with cetuximab plus irinotecan which have been explored in the SWOG 1406 trial with encouraging results^[61]^. Moreover, several other promising strategies designed to overcome resistance pathways to *BRAF*-inhibitors are currently under investigation^[62,63]^. Final results from ongoing trials are warranted to improve targeted treatment options for *BRAF*-mutated patients.

### Microsatellite Instability

MMR is a highly conserved DNA repair mechanism that ensures genomic integrity by correcting mispaired or unpaired bases which have escaped the proofreading activity of DNA polymerases during DNA replication and recombination, as well as repairing some forms of DNA damage. The loss of MMR proteins activity leads to an accumulation of DNA replication errors, a phenomenon known as MSI, characterized by high frequency of frameshift mutations in microsatellite DNA which translates into a high somatic mutational burden in MMR-deficient (MMR-D) cells (mutator phenotype)^[64]^.

The prevalence of MSI in CRC depends on the stage of the disease. Approximately 20% of CRCs in stage I-II, 12% in stage III and 4%−5% in stage IV, are deficient in one or more DNA MMR proteins, with one-quarter of these resulting from Lynch syndrome (LS), an autosomal dominant condition characterized by germline mutations in genes coding for MMR proteins (i.e. *MLH1*, *MSH2*, *MSH6*, *PMS2* or *EPCAM*)^[65]^. The vast majority (circa 80%−90%) of sporadic MSI cases are due to hypermethylation of the *MLH1* gene promoter^[66,67]^, associated with a high CpG island methylation phenotype (CIMP+) and about 30% harbor a *BRAF* V600E mutation^[6,68]^. The remaining cases of sporadic MSI can be explained mainly by the presence of multiple somatic mutations in the MMR genes without an identifiable germline MMR mutation (“double somatic” MSI cases)^[69]^, found to be associated with a higher frequency of somatic mutations in *PIK3CA*^[70]^. According to the recent CMS classification MSI is associated with CMS1^[6,71]^. MSI detection is currently based on two different approaches: immunohistochemical staining (IHC) for MLH1, MSH2, MSH6, and PMS2 on tumor samples to identify the loss of protein expression which characterizes MMR deficiency as a surrogate for MSI^[72]^; DNA MSI testing through a polymerase chain reaction (PCR)-based approach evaluating specific panels of microsatellite markers^[73]^. If either MSI or MMR deficiency is detected, further evaluation is recommended to rule out LS, rather than sporadic MSI. Of note, recently new computational approaches based on the evaluation of next generation sequencing (NGS) data have been proposed as a tool for MSI assessment^[74–77]^, as well as the evaluation of mutational burden on circulating cell-free tumor-DNA testing as a surrogate marker of mismatch repair deficiency or microsatellite instability in patients with CRC^[78]^.

MSI-H CRCs are characterized by distinct clinical and pathological features such as right-sided colon location, early-stage at diagnosis, prominent lymphocytic infiltrate, poor differentiation and mucinous histology^[79]^. When diagnosed in the metastatic setting, MSI-H mCRCs arise more frequently in women and in elderly; presenting often with synchronous metastases involving peritoneum, lymph nodes and lung rather than liver. Notably, distinct patterns characterize inherited and sporadic MSI-H mCRCs^[80]^. In addition to LS screening, in patients with early-stage (especially stage II) CRCs, MMR status provides important prognostic and predictive information, with MMR deficiency being associated with both a good prognosis and apparently a lack of efficacy from fluorouracil treatment, although data regarding whether or not MSI status predicts response to adjuvant chemotherapy in this setting has been controversial^[81–85]^. The most solid data derive from the analyses of the ACCENT database investigating the impact of MSI in stage II and III CRCs treated with surgery *vs.* surgery followed by 5-FU-based adjuvant therapy across 17 different trials. Stage II and III patients with MSI tumors showed better outcome with surgery alone compared to those with microsatellite stable (MSS) tumors. Conversely, stage III patients showed a significant survival benefit from the addition of 5-FU adjuvant therapy after surgery both in case of MSS and MSI tumors^[84]^. To date, adjuvant chemotherapy is not recommended for patients with low risk stage II MSI-H tumors due to their excellent prognosis, while stage III patients should receive adjuvant treatment irrespective of MSI status. Of note, MSI etiology (germline *vs.* sporadic) seems to affect the predicted benefit from 5-FU, as Sinicrope *et al.*^[86]^ showed, in a retrospective evaluation of stage II and III CRC patients who received either adjuvant 5-FU or placebo, that individuals with MSI-H CRCs due to germline mutations (i.e. LS) had an improved disease free survival (DFS) with 5-FU compared to those with sporadic MSI-H tumors. The role of MSI as a predictive marker with modern combination regimens, such as FOLFOX and FOLFIRI, has less evidence^[87–89]^, and although an MSI-H status was retrospectively shown to predict improved DFS with adjuvant irinotecan and 5-FU (IFL regimen) in the CALGB (Alliance) 89803 trial, these results were inconsistently demonstrated in other exploratory analyses^[90,91]^. In the metastatic setting, recent data suggest a greater activity of irinotecan in MSI-H mCRC and better outcomes in favor of bevacizumab treatment compared to anti-EGFRs^[92]^. Indeed, vascular endothelial growth factor (VEGF) is known to play a crucial role in tumor microenvironment immuno-modulation and anti-angiogenic treatment has been proposed as an effective modality to potentiate immunotherapy^[93]^. No definitive evidence is available on the prognostic role of MSI-H in mCRC; recent data suggest no statistically significant difference in OS between MSI-H and MSS mCRCs, although a trend toward a worse OS has been reported for MSI-H^[94]^. Some studies suggest the correlation with *BRAF* mutational status as a potential confounding factor affecting the estimation of MSI-H impact on survival in mCRC^[95]^. However, the prognostic role of *BRAF* in these tumors is still object of debate and in a recent analysis *BRAF* V600E mutation was not associated with a worse survival in MSI-H CRC^[80]^. Additionally, a possible negative prognostic effect of immune checkpoint expression in MSI-H CRCs have been recently reported, which seems to be able to counterbalance the positive effect of tumor-infiltrating cytotoxic T-cell lymphocytes in these tumors^[96]^.

MSI assessment has lately gained a prominent role in the metastatic setting due to the recent groundbreaking success of immunotherapy with checkpoint inhibitors in MMR-D mCRCs which has opened a new era in the treatment of MSI-H tumors. In the phase II KEYNOTE 016 trial, pembrolizumab demonstrate its activity in 28 MSI-H mCRC patients with refractory disease, significantly improving response rate (RR), disease control rate (DCR), median PFS and OS compared to MSS patients (RR: 50% *vs.* 0% and DCR 89% *vs.* 16%, respectively; HR for PFS = 0.135, *P* < 0.001, HR for OS = 0.247, *P* = 0.001)^[97,98]^. The combination of ipilimumab (an anti-CTLA4) and nivolumab (an anti-PD1), under investigation in the phase II CHEKMATE142 trial, showed as well significant results with a recently reported RR of 31.1% (95% CI, 20.8–42.9) in patients receiving nivolumab (*n* = 74) and 55% (95% CI, 45.2–63.8) in those receiving ipilimumab plus nivolumab (*n* = 119), and remarkable 12 months PFS rate and 12 months survival rate (50% and 73% respectively, for nivolumab monotherapy; 71% and 85% respectively, for nivolumab plus ipilimumab)^[99,100]^. Responses were irrespective of tumor *RAS* and *BRAF* mutational status, immune cell PD-L1 expression or clinical history of LS. Notably, both pembrolizumab and ipilimumab/nivolumab showed a trend towards a plateau in the tail of patients’ survival curves, suggesting the possibility of long term responders similar to the previous experience with immunotherapy in melanoma. Following these striking results, FDA approval was granted for the use of checkpoint inhibitors pembrolizumab (Keytruda®, Merck & Co., Inc.)^[101]^ and nivolumab (Opdivo®, Bristol-Myers Squibb)^[100]^ in the treatment of MSI-H or MMR-D mCRC.

Despite the clinical success of anti-CTLA4 and PD-L1/PD-1 inhibitors, however, only a subset of selected patients exhibits durable responses, suggesting that a broader view of cancer immunity is required. A complex set of dynamic tumor, host and environmental factors modulate the strength and timing of immune anticancer response, and several key immunoregulatory pathways have been identified and involved in the definition of an immune signature to predict responses to immunotherapy^[102–105]^. Alongside the ongoing extensive effort to identify additional predictive biomarkers^[106,107]^, understanding the mechanisms limiting immunotherapy efficacy, both in terms of innate and acquired resistance, represents a challenge which needs to be addressed in order to improve treatment outcomes and develop new actionable strategies^[108–110]^.

### Dihydropyrimidine dehydrogenase

Fluoropyrimidine analog 5-FU and its pro-drug capecitabine represent the backbone of chemotherapy treatment for colorectal cancer^[10]^. The mechanism of action of these drugs is based on thymidylate synthase (TYMS) inhibition through the formation of a ternary complex between the active metabolite 5-fluoro-2-deoxyuridine-5-monophosphate (5-FdUMP), TYMS and 5,10-methylentetrahydrofolate, leading to the suppression of DNA synthesis^[111]^. The rate-limiting enzyme for 5-FU catabolism is the enzyme dihydropyrimidine dehydrogenase (DPD), responsible for the inactivation of more than 80% of the administered dose of 5-FU^[112]^.

Up to one-third of patients treated with these agents experience severe (and in 0.5%−1% of cases lethal) toxicities including myelosuppression, mucositis and diarrhea^[113]^. Functional DPD gene (*DPYD*) variants leading to a decreased enzymatic activity have been found to correlate with the risk of 5-FU and capecitabine severe toxicities in several pharmacogenetic studies. Over 30 single nucleotide polymorphisms (SNPs) in the *DPYD* gene have been studied over the last 20 years, although many of these variants did not appear to have any functional effect. Among the most well-known, the c.2846 A>T and c.1679 T>G variants, alongside the G>A mutation (DPYD*2A) of the invariant splice site in exon 14 (IVS14+1G>A), coding for a truncated protein with no enzymatic activity, have been consistently associated with decreased DPD activity and a 4-fold increase of risk of developing 5-FU related toxicities^[114]^. DPYD*2A is the most frequent SNPs in the Caucasian population, nevertheless its incidence is low (about 1%−2% for the heterozygote genotype) and shows substantial ethnic variations. Homozygous for DPYD*2A have been associated with cases of lethal toxicities in patients treated with fluoropyrimidine-based chemotherapy^[115,116]^. More recently a large meta-analysis from Meulendijks *et al.*^[117]^ confirmed the predictive role for drug-related toxicities for four *DPYD* variants: DPYD*2A, c.2846A>T, c.1679 T>G and c.1236G>A/haplotype B3. Data from retrospective pharmacogenetic analyses from the Italian adjuvant TOSCA trial confirm the role of DPDY*2A as a risk factor for fluoropyrimidine-related toxicities^[118]^. Additionally, a prospective study enrolling 2,038 patients candidate to receive a fluoropyrimidine-based chemotherapy demonstrated the feasibility and cost-effectiveness of upfront DPYD*2A genotyping before treatment start. DPYD*2A variant allele carriers were treated with a reduced dose-intensity leading to a significant reduction of the risk of grade ≥ 3 toxicity (28% *vs.* 73% in historical controls, *P* < 0.001) and a reduction of drug-induced death from 10% to 0%^[119]^. The low frequencies of the aforementioned risk alleles, however, cannot fully explain the estimated risk of DPD-linked fluoropyrimidine-related adverse events, underlining the complex multi-level modulation of DPD activity, involving both transcriptional and post-transcriptional mediators, and the need to investigate additional *DPYD* risk variants. Nevertheless, available data support the role of *DPYD* testing as a pre-treatment screening in patients undergoing 5-FU and capecitabine treatment in order to improve the safety of fluoropyrimidine-based therapies and potentially allow genotype-guided dose adaptations, as recently recommended by the clinical pharmacogenetics implementation consortium^[120]^.

Evidence on the role of DPD deficiency as a toxicity biomarker led the FDA to include a warning annotation on the label of fluorouracil for patients with low or absent DPD activity, recommending to withheld or permanently discontinue fluorouracil in patients with evidence of acute early-onset or unusually severe toxicity, which may indicate near complete or total absence of DPD activity. On the other hand, latest published ESMO clinical practice guidelines on metastatic colorectal cancer management suggest for the first-time pre-treatment DPYD testing as an option^[9]^. This indication, however, is focused on those patients who experience severe 5-FU toxicity before 5-FU re-introduction and routine testing is not recommended, despite the authors stating that patients with known partial DPD deficiency benefit from dose adaptation of 5-FU/capecitabine therapy to avoid severe toxicity, while in patients with complete DPD deficiency fluoropyrimidines should be avoided and an alternative treatment offered. The lack of recommended standardized assessment techniques represents an additional issue to the introduction of routine DPD testing.

The predictive role of genetic variants in other key genes involved in the folate pathway, such as TYMS and 5,10-methylenetetrahydrofolate reductase, has not been validated and their use in clinical practice is not recommended.

### UDP-Glucuronosyltrasferase A1

Irinotecan, a topoisomerase I inhibitor, is another key drug in the chemotherapy treatment of mCRC, which can be used as a monotherapy or in combination with 5-FU and/or other agents in different treatment lines^[9,10]^. This agent is administered as a pro-drug which is metabolized to its active form, SN-38, via carboxylation. SN-38 catabolism and excretion are subsequently dependent on conversion to its inactive form, SN-38G, operated by hepatic UDP-Glucuronosyltrasferases (UGT) such as UGT1A1^[121]^. Additionally, the pharmacokinetics of irinotecan involves several other enzymes, such as CYP3A4, which control its metabolism modulating the available dose of the active drug. A genetic variation in these enzymes can affect tolerability and toxicity profile in patients.

Up to 36% of patients treated with irinotecan-containing regimens experience severe and potentially life-threatening adverse events, such as neutropenia and diarrhea^[122]^. Variations in the UGT1A1 activity have been shown to be associated with irinotecan-induced toxicities. The most common gene variants are the UGT1A1 *1 and *28 alleles, representing 98%−99% of all variants in the Caucasian population. The *28 variant, responsible for Gilbert syndrome, is characterized by the presence of an extra TA repeat in the promoter of the *UGT1A1* gene which is associated with a remarkably reduced enzymatic activity and correlates with higher incidence of drug-related adverse events due to a slower catabolism of SN-38G^[123]^. In USA, about 45% of the population is heterozygous for the *28 allele (*1/*28) while around 10% carries a homozygous genotype for this variant. The frequency increases in the African population and is lower in South-East Asian and Pacific populations. The role of UGT1A1 genotyping has been evaluated in several clinical trials, and two large meta-analyses including nearly 2000 patients confirmed that carriers of the UGT1A1 *28/*28 genotype were at a higher risk for neutropenia compared to WT *1 patients even at a low irinotecan dosage (80–145 mg/m^2^)^[124]^, while carriers of the *28 allele were at risk of severe diarrhea at doses above 125 mg/m^2^^[125]^. Consistently, genotyping analyses of patients treated with 5-FU and irinotecan within the randomized phase III Nordic IV trial^[126]^ and the randomized phase III TRIBE trial^[127]^, confirmed the association between the UGT1A1*28/*28 genotype and higher risk of neutropenia. Subsequent meta-analyses most recently supported once again the role of UGT1A1*28 as predictive of irinotecan-related severe toxicities, as well as the role of additional variants such as UGT1A1*6, a missense variant frequent in the Asian population^[128,129]^. Finally, a recent dose-finding and pharmacokinetic study suggests that irinotecan treatment dose should be individualized based on UGT1A1 genotype. Results from this study, in fact, show that the maximum tolerated dose of irinotecan, administered as an intravenous infusion every 3 weeks, was 850, 700, and 400 mg in patients bearing the *1/*1, *1/*/28, and *28/*28 genotypes, respectively^[130]^.

Based on available data the latest ESMO guidelines suggest UGT genotyping as an option in patients with a suspicion of UGT1A1 deficiency and when the administration of a dose of irinotecan >180 mg/m^2^ is planned^[9]^. On the other hand, the National Comprehensive Cancer Network guidelines version 2.2017 states that irinotecan should be used with caution and at a decreased dose in patients with Gilbert syndrome or elevated serum bilirubin, but routine genotyping of UGT SNPs is not recommended^[10]^. It has to be noted, however, that FDA has modified irinotecan label to include a toxicity warning for the UGT1A1*28 polymorphism, suggesting an initial dose reduction when treating patients carrying the UGT1A1*28 homozygous allele.

## EMERGING BIOMARKERS OF SPECIAL INTEREST HER2

Although tumor *RAS* WT status is, as previously described, a crucial prerequisite for anti-EGFRs activity in mCRC, several patients with *RAS* and *BRAF* WT tumors still do not benefit from anti-EGFR treatment. Based on preclinical data and retrospective evaluations, additional mechanisms of primary resistance to anti-EGFR agents have been identified over time in *RAS* WT mCRC, including human epidermal growth factor receptor 2 (*HER2/neu*) amplification. HER2 is a member of the EGRF family which regulates key cellular processes such as proliferation and apoptosis through the activation of the RAS/RAF/ERK and the PI3K/PTEN/AKT signalling pathways. *HER2* role as a driver oncogene in CRC and as potential biomarker for targeted treatment in the metastatic setting has recently been the object of great interest.

First data were reported in 2011 when *HER2* amplification (which can be found in approximatively 5% of *RAS* WT mCRCs), was detected in a subset of *KRAS/NRAS/BRAF/PIK3CA WT* cetuximab-resistant patient-derived xenografts. Following this first evidence, a proof-of-concept study in the subgroup of *HER2*-amplified xeno-patients demonstrated a significant tumor regression after combined treatment with HER2 and EGFR blockade^[131]^. These results were subsequently challenged in an Italian phase II clinical trial, the HERACLES study. More than 1000 mCRC cases were analysed in order to identify strict criteria for the definition of *HER2* amplification^[132]^ in the dedicated HERACLES diagnostic. Afterwards, the activity of an HER2 double blockade with trastuzumab and lapatinib was evaluated in chemorefractory mCRC patients with *HER2*-positive tumors. Initial results of the study have been published, showing a 30% objective response rate (95% CI, 14–50), with one patient achieving a complete response, and a 44% stable disease rate (95% CI, 25–63)^[133]^. Of note, none of the 15 patients (56%) evaluable for response to anti-EGFRs achieved an objective response to previous treatment with either cetuximab or panitumumab, supporting the role of *HER2* amplification as a mechanism of primary resistance to anti-EGFR targeted agents. Moving from such promising results, a second cohort of the study has enrolled patients to treatment with a combination of trastuzumab-emtansine (TDM1) and pertuzumab, and patients experiencing disease progression after treatment with trastuzumab and lapatinib are receiving TDM1 monotherapy within the HERACLES Rescue trial. New results from these studies are highly anticipated.

Confirmatory results on HER2 as a possible target in mCRC came also from the phase II MyPathway trial, and retrospective series confirmed data on *HER2* as a possible predictive biomarker of resistance to anti-EGFRs^[134]^. Additionally, *HER2* amplification detected on tissue or on circulating tumor DNA (ctDNA) was identified as a possible mechanism of acquired resistance in *HER2* negative, *RAS/BRAF* WT, patients progressed during anti-EGFR treatment^[135]^. Of note, a randomized phase II trial, the S1613 study, has been recently opened to explore the efficacy of trastuzumab and pertuzumab compared to cetuximab and irinotecan in pre-treated anti-EGFR naïve mCRC patients carrying a tumor with *HER2/neu* amplification^[136]^.

Supported by a strong preclinical rationale and confirmatory clinical data *HER2* testing might be soon implemented in clinical practice for patients with mCRC candidate to receive anti-EGFR and/or anti-HER2 treatments.

### Anti-EGFR agents: other biomarkers of primary and acquired resistance

Alongside *HER2* amplification, several other mechanisms of primary resistance to anti-EGFR targeted treatment have been identified so far, including phosphatidylinositol-3-kinasecatalytic subunit alpha (*PIK3CA*) mutations (exon 9 and 20 hotspot mutations), *MET* amplification, *FGFR1* and *PDGFRA* mutations, loss of *PTEN* function and low *EGFR* copy number^[137]^. However, the routine use of these biomarkers in clinical practice cannot be recommended at present, and further prospective validation of their predictive role is warranted. Nevertheless, different combined strategies and novel targeted agents aimed to overcome primary resistance to anti-EGFRs are currently under investigation, such as the combination of anti-EGFR agents with mammalian target of rapamycin (mTOR) inhibitors^[138]^. Recently, a panel of genomic alterations (the PRESSING panel) comprising activating mutations of the MAPKs or PI3K/AKT axis, *HER2* amplification or mutations, *MET* amplification and *NTRK/ROS1/ALK/RET* rearrangements, have been tested in an interesting retrospective case-control study aiming to dissect primary resistance to anti-EGFR treatment, demonstrating the negative predictive impact of these mutations in *RAS/BRAF* WT mCRCs treated with anti-EGFRs^[139]^. The study included 47 cases (patients resistant to anti-EGFR-containing regimens) and 47 controls (patients who responded to single agent anti-EGFRs or to a combination of irinotecan with anti-EGFRs if previously clearly irinotecan refractory). Aforementioned genomic alterations were reported in 20 (42.6%) cases and 1 (2.1%) control (*P* < 0.001), meeting the primary endpoint of the study. Additionally, primary tumor right-sidedness was found to be associated with resistance to anti-EGFRs, confirming recent literature evidence, and the combined evaluation of PRESSING panel and primary tumor location demonstrated the best predictive accuracy. These results open promising perspectives on the clinical application of a more comprehensive molecular characterization of *RAS/BRAF* WT mCRCs to further improve and refine patients selection.

Secondary resistance to anti-EGFRs is often dependent on clonal selection induced by targeted treatment pressure. Emerging mutations in the RAS/RAF/MAPK signaling pathway can be detected after disease progression in tumor biopsies from previously *KRAS* wild-type tumors and multiple mutations can coexist at the same time in the same sample^[140]^. This seems to be the result of the amplification of pre-existing minor sub-clones, suggested by a significant overlap in the genetic events associated with primary and acquired resistance^[141]^. Moving from these data, several trials are currently exploring different approaches to multiple targeted inhibition based on the emergence of selected resistance drivers, such as the combination of anti-EGFRs with MEK or MET inhibitors. Mutations in the ectodomain of EGFR represent an additional mechanism of resistance limited to the acquired setting^[142,143]^. Notably, a subset of mutations including *EGFR* S492R as well as other acquired mutations recently identified (S464L, G465R and I491M) appears to confer resistance to cetuximab but not panitumumab. The binding epitopes of cetuximab and panitumumab on EGFR, in fact, overlap but are not identical^[144,145]^. Retrospective analyses from the ASPECCT trial, comparing panitumumab to cetuximab in chemorefractory mCRC patients, revealed that EGFR S492R mutations occurred in 1% *vs.* 16% of patients treated with panitumumab and cetuximab, respectively^[146]^. The possible rationale for using panitumumab after the detection of these mutations as a mechanism of resistance to cetuximab still need further validation. Other strategies to overcome acquired resistance to anti-EGFRs include treatment with novel antibodies targeting different epitopes of the EGFR ectodomain, which can increase receptor internalization and degradation such as MM-151^[147]^ and Sym004^[148]^.

### VEGF pathway

Angiogenesis plays a key role in CRC development and progression, and VEGF is a key regulator in both physiological and pathological angiogenesis. Therapeutic agents targeting VEGF/VEGFR signaling (i.e. bevacizumab, aflibercept, ramucirumab and regorafenib) proved to be effective across different treatment lines in mCRC and contributed greatly to improve patients’ survival in recent years^[9,10]^. However, despite extensive efforts to identify predictive biomarkers for antiangiogenic therapies in the last decade, no predictive marker is available in clinical practice yet^[149]^. The complexity of the angiogenesis signaling network and the overlap between various angiogenic factors, in fact, represent a challenge to pharmacogenomic biomarkers discovery.

In 2012, Bates *et al.*^[150]^ retrospectively analyzed CRC tumor samples from the phase III bevacizumab E3200 trial to explore the predictive value on treatment outcomes of VEGF165b, a VEGF splice isoform. Despite not reaching a statistical significance, patients with a lower level of VEGF165b appeared to benefit more from bevacizumab treatment. Focusing on a different candidate marker, recently published data demonstrated that patients treated with first-line bevacizumab-containing regimens had a significantly longer PFS when affected by *Homeobox B9* (*HOXB9*)-negative tumors compared with those with *HOXB9*-positive tumors (18.0 *vs.* 10.4 months, *P* = 0.048). *HOXB9* is known as a highly conserved homeobox transcription factor gene which drives neoplastic transformation and tumor progression exerting an anti-apoptotic effect and promoting tumor cell invasion. The authors demonstrated, both with preclinical and clinical data, that transcription factor HOXB9 mediates resistance of CRC to bevacizumab modulating a complex network of alternative pro-angiogenic and pro-inflammatory secreted factors^[151]^. A prospective validation of these promising results is highly anticipated. In another interesting analysis, NOTCH1 expression has been proposed as a detrimental prognostic factor in mCRC patients treated with chemotherapy plus bevacizumab^[152]^. Of note, a phase Ib trial is ongoing exploring safety and preliminary efficacy of a bispecific antibody targeting VEGF and the NOTCH ligand DLL4 (OMP-305B83) in combination with FOLFIRI as second-line treatment in mCRC^[153]^. Finally, a novel emerging player in the angiogenesis regulatory pathways is the protein apelin (APLN). APLN signaling takes part in multiple physiological functions including angiogenesis, and interacts at different levels with key mechanisms regulating cell growth, survival and apoptosis. Recent preclinical data based on the analysis of tumor-derived endothelial cells from patients receiving bevacizumab showed that APLN mRNA levels are significantly associated with treatment response. In fact, APLN levels were high in non-responders and low in patients who benefitted from bevacizumab (*P* = 0.0001)^[154]^. All these potential biomarkers, however, still need validation.

As novel anti-angiogenic agents have entered clinical practice in recent years, the interest was directed to identify specific biomarkers for each compound. A retrospective analysis of ctDNA from liquid biopsies collected from about 350 patients treated with regorafenib in the CORRECT trial was performed to investigate the impact of *KRAS*, *PIK3CA* and *BRAF* mutations on regorafenib efficacy. Results were consistent with previous data and confirmed that the benefit from regorafenib on survival and treatment outcomes was irrespective of *KRAS* and *PIK3CA* mutational status^[155]^. The analysis according to *BRAF* mutational status, on the other hand, was not feasible due to the small number of *BRAF*-mutated patients. Data on *RAS*, *BRAF* and sidedness as biomarkers in patients treated with aflibercept in the VELOUR trial have been recently presented as well. No significant interactions according to *RAS* and *BRAF* status were found in this analysis, although a trend for better outcomes was observed for *BRAF*-mutated tumors treated with aflibercept in comparison with the control arm (mOS 10.3 *vs.* 5.5 months, respectively, HR 0.42; 95% CI, 0.16–1.09; *P* = 0.08)^[156]^. Similar results were observed in patients treated with ramucirumab within the RAISE trial. In fact, the ramucirumab favorable treatment effect was similar between *RAS*-mutated and all *RAS*/*RAF* WT tumors; however, the benefit was more notable in *BRAF*-mutated tumors both for OS (HR 0.54; 95% CI 0.25–1.13) and PFS (HR 0.55; 95% CI 0.28–1.08)^[157]^. Additionally, Tabernero *et al.*^[158]^ assessed the correlations of a series of baseline marker levels (including VEGFR-2 immunohistochemistry in tumor tissue) with clinical outcomes in the RAISE patients population. Only VEGF-D circulating serum levels were found to be statistically significant with higher levels of this soluble factor (≥ 115 pg/mL) associated with improved ramucirumab efficacy in comparison with placebo^[158]^.

Several SNPs in different genes involved in VEGF signaling pathway have been investigated over time. Results from a large meta-analysis including 158 SNPs and 1348 patients enrolled in five phase III randomized trials suggested an association between VEGFA rs699946 and VEGFR-2 rs11133360 polymorphisms and improved PFS in bevacizumab-treated patients^[159]^. Unfortunately, additional promising retrospective findings on different candidate SNPs of VEGF/VEGFR pathway genes were not prospectively validated in a dedicated study^[160]^.

### DNA methylation

Over the last decade, evidence on the role of the epigenome in CRC has been largely explored and it is now recognized that among thousands of epigenetic alterations which can be present in each tumor, a small subgroup may be considered a driver event in CRC development^[161]^. Different epigenetic mechanisms, in fact, can play a key role in carcinogenesis, such as DNA methylation, nucleosome positioning, histone modifications and non-coding RNAs expression^[162]^. Technological advances have considerably increased our ability to detect a wide number of epigenetic alterations which can eventually have a role as clinical biomarkers for early detection, prognostic stratification and treatment efficacy prediction in CRC patients. Of note, recently the availability of more refined genome-wide mapping technologies, highlighted that the function of DNA methylation can vary depending on its context, underlining a deep complexity that warrants further evaluations^[163]^.

Aberrant DNA methylation is the most extensively studied epigenetic mechanism in CRC. Global DNA hypomethylation is currently considered a common feature of CRC; on the other hand, however, evidence on the role of CpG islands DNA hypermethylation in promoting CRC by silencing the expression of tumor suppressor genes led to the identification of the CpG Island Methylator Phenotype (CIMP), consisting in a subset of CRCs characterized by distinct epidemiological, histological and molecular features and prognosis^[164]^. CIMP+ tumors are associated with female gender and older age, show more frequently a right-sided colon location, a high incidence of BRAF V600E mutation and MSI-H status as a consequence of *MLH1* epigenetic silencing through promoter DNA hypermethylation, diploid copy number and absence of TP53^[165]^. CIMP status has been proposed as a promising prognostic marker for CRCs, however, several studies reported contradictory results, possibly due to the overlap between the CIMP+ phenotype and the MSI-H phenotype, associated in 30%−50% of cases with *BRAF* mutation^[166]^. The lack of global consensus in defining CIMP+ tumors, together with these controversial results, has hindered the uptake of CIMP as a relevant biomarker in clinical practice and further studies are warranted to explore its predictive and prognostic value^[167]^.

Long interspersed nucleotide element-1 (LINE-1) methylation measured by pyrosequencing has been shown to correlate with global DNA methylation levels^[168]^. LINE-1 is a retrotransposon related to key CRC features involved in the carcinogenesis process: LINE1 hypomethylation is associated with 18q loss of heterozygosity (LOH); whereas an inverse correlation has been demonstrated between LINE-1 hypomethylation, CIMP-H and MSI-H status. LINE-1 methylation levels have been reported to impact CRC prognosis with hypomethylation conferring poor prognosis in terms of overall mortality (OM) and colorectal cancer-specific mortality^[169]^. Additionally, LINE-1 hypomethylation in MSS/CIMP+ stage II and III CRC has been showed to predict benefit from adjuvant chemotherapy with oral fluoropyrimidines^[170]^. These data suggest that DNA demethylation may play, as well, a crucial role in CRC development, prognosis and response to treatment. Although promising, however, these findings need further validation.

The DNA repair gene O6-methylguanine-DNA methyltransferase (*MGMT*) has recently gained attention and has been object of several studies. This gene encodes a DNA repair protein which removes alkylating groups from O6-guanine and is involved in protecting cells against damages from alkylating agents. *MGMT* has been shown to undergo epigenetic silencing by promoter hypermethylation in more than 40% of mCRCs^[171]^. The loss of *MGMT* gene expression impairs the ability of DNA repair mechanisms to remove alkyl groups, potentially enhancing the cytotoxic effects of alkylating drugs, such as dacarbazine and temozolomide. On these bases, several phase II clinical trials^[172]^ evaluating the efficacy of alkylating agents in mCRC have been conducted with promising results. In these studies, MGMT methylation has been used as a predictive biomarker for patients’ selection, supporting a possible role for this novel marker in clinical practice.

In an era in which immuno-oncology is revolutionizing cancer treatment strategies, novel possible relevant implications of aberrant DNA methylation come from its tight connection with the immune cells system. To date, immune-checkpoint inhibitors (ICI) have shown striking results in selected cancer types, although only a minority of patients are sensitive to these drugs. *De novo* DNA methylation has been recently reported to have a central role in maintaining a T cell exhaustion status that contributes to resistance to ICI treatment^[173]^. On the other hand, previous studies demonstrated that DNA demethylating drugs can enhance CTLA-4 blockade-mediated T cell responses^[174]^. Moreover, treatment of epithelial cancer cell lines (including CRC cell lines) with demethylating agents, i.e. 5-azacitidine, has been reported to promote a significant enrichment of immunomodulatory pathways^[175]^. As a possible explanation, cryptic transcription of thousands of treatment-induced non-annotated transcriptional start sites (TINATs) may contribute to cancer immunogenicity through the translation of novel potential antigenic proteins, as recently shown by Brocks and colleagues in their work exploring DNA methyltransferases inhibitors (DNMTi) treatment consequences on epigenetic and genome-wide transcription^[176]^. Overall, this growing evidence supports a strong immunomodulatory effect of DNA demethylating agents in cancer cells, and the rationale to combine these drugs with immunotherapy in cancer patients. Based on these premises, a deeper understanding of the interplay between epigenetic modifications, cancer cells and immune cells could reveal novel potential strategies to enhance ICI treatment efficacy and overcome primary and acquired resistance mechanisms to immunotherapy.

Finally, aberrant DNA methylation may exert a direct effect modulating well-established molecular pathways in CRC. Notably, *EGFR* promoter DNA methylation has been reported to occur in 58% of primary colon tumors and to be strongly correlated with shorter patients’ PFS and OS (PFS 2.4 *vs.* 7.4 months, *P* < 0.0001; OS 6.1 *vs.* 17.8 months, *P* < 0.0001)^[177]^. On the other hand, Khambata-Ford *et al.*^[178]^ discovered that patients with overexpression of epiregulin (EREG) and amphiregulin (AREG), two EGFR ligands, are more likely to achieve disease control when treated with cetuximab and show a significantly longer PFS. These data have been confirmed by Jacobs *et al.*^[179]^ showing a significant association between cetuximab response and AREG/EREG expression. In a recent work, EREG and AREG expression has been found to have a strong inverse correlation with methylation and to be inversely associated with right-sided tumor location, CIMP-H status and BRAF mutation^[180]^. Additionally, the authors reported that treatment with hypomethylating agents (i.e. azacitidine) increased EREG expression, and that a CIMP-H status was associated with shorter PFS outcomes, also in *BRAF*/*NRAS* WT patients. Based on these data, promoter DNA methylation may be the main regulatory mechanism of AREG/EREG expression, which may explain, at least in part, the association between right-sided tumor location, CIMP-status and anti-EGFR treatment response in mCRC. DNA methylation may, then, partially account for primary anti-EGFRs resistance, supporting the rationale to explore the possible synergistic treatment effect of demethylating agents in combination with anti-EGFR drugs.

Despite promising evidence, the complexity and heterogeneity of epigenetic alterations in CRC still represent a considerable challenge, which needs to be further addressed in order to identify reliable biomarkers and translate current knowledge into actionable therapeutic strategies.

## FUTURE PERSPECTIVES

### CRC consensus molecular subtypes

In recent years, great advances have been made in understanding the complexity of tumor biology and genetic landscape underlying tumor development and response to treatment. In 2015 an international consortium developed the Consensus Molecular Subtypes, which classifies CRC into four distinct biological groups, based on gene expression signatures and correlated with distinct genetic, epigenomic, transcriptomic, microenvironmental, prognostic and clinical features^[181]^. CMS1 (microsatellite instability immune, 14%) tumors are associated with high tumor mutational load (TML), microsatellite instability, hypermethylation status (CIMP+), *BRAF* mutation, and strong immune activation. The CMS2 (canonical, 37%) subtype is characterized by an epithelial signature, marked WNT-β-catenin pathway and MYC signaling activation. CMS3 (metabolic, 13%) tumors feature metabolic dysregulation; and CMS4 (mesenchymal, 23%) a prominent transforming growth factor (TGF)-β activation, stromal invasion and angiogenesis. Samples with mixed features (13%) are considered to represent a transition phenotype or intratumoral heterogeneity. CMS subgroups show a strong prognostic value independent of tumor stage, with CMS4 associated with worse survival. Moreover, retrospective analyses of clinical trials have suggested a potential predictive value for CMS subtypes, including a better outcome following bevacizumab treatment for CMS1^[182]^, and a lack of benefit from oxaliplatin^[183]^ and anti-EGFRs (irrespective of *RAS* mutational status)^[184]^ for the mesenchymal-like phenotype. Although not yet implemented in clinical practice, this classification system has the potential to better inform clinicians of prognosis and therapeutic response, and to guide novel therapeutic strategies with subtype-based targeted interventions^[6]^. In fact, data have been published from very recent preclinical studies exploring models of CMS in large panels of CRC cell lines, primary cultures and patient-derived xenografts (PDX), with the aim of developing “adapted” classifiers optimized for pre-clinical research and investigate specific drug sensitivity of individual CMS^[185,186]^. Results from these studies show interesting initial findings highlighting subtype-dependent response profiles, with a different sensitivity to chemotherapy (either 5-FU or oxaliplatin)-induced apoptosis between CMS2 and CMS4, which relates to the *in vivo* efficacy of chemotherapy in PDX models where a delay in outgrowth of CMS2, but not CMS4 xenografts, was observed. Additionally, a strong response to anti-EGFRs and HER2 inhibitors was observed in the CMS2 subtype. Indeed, a deeper understanding of the unique drug-sensitivity profile of each CMS subtype and the possibility of performing high-throughput *in vitro* and *in vivo* drug screening using PDX technology have the potential to greatly advance precision medicine in CRC.

### Liquid biopsy

Another field of major interest is the rapid development of liquid biopsies technology and the analysis of ctDNA as a more comprehensive and less invasive approach to pharmacogenomic profiling in CRC patients^[187,188]^. Allowing large-scale genomic profiling and being able to capture the molecular heterogeneity of different tumor sub-clones coexisting in the same patients, these techniques are expected to play a pivotal role in improving patients stratification and selection for targeted treatments. Moreover, the possibility to perform seriated testing over time represents a valid opportunity to guide treatment strategies through an early detection of the emergence of treatment resistance and a dynamic tumor molecular profiling^[189]^. Indeed, data from repeated ctDNA analyses have been able to show the emergence of *RAS* and/or *BRAF* mutations during treatment with anti-EGFRs in *KRAS* WT patients, closely dependent on treatment exposure, with a dynamic increase during EGFR blockade followed by a rapid decline after treatment withdrawal^[190]^. Recently, a large study on genomic profiling through liquid biopsy analyzing next generation sequencing data from cell-free DNA of 1397 CRC patients, confirmed the reliability of this methodology in detecting genomic alterations when compared with corresponding tissue-based sequencing. Additionally, results of this study highlighted the possibility of detecting the development of multiple distinct concomitant mechanisms of resistance after targeted treatment with anti-EGFRs in the same subject, proving that ctDNA sequencing can generate a valuable insight into tumor heterogeneity and therapeutic resistance^[191]^. Although still needing extensive investigations and prospective validation, liquid biopsy approaches to profile tumor dynamics and response to treatment and to guide rechallenge strategies based on detection of circulating genomic alterations are currently under investigation in several clinical trials.

### MiRNAs

Finally, noncoding RNAs represent an evolving field in cancer diagnosis and prognosis, and several studies have suggested their possible role as treatment target in different diseases^[192,193]^. miRNAs are noncoding single-stranded RNA molecules, less than 200 nucleotides in length, with a post-transcriptional regulatory function involved in the modulation of a broad range of biological processes comprising cellular signaling, metabolism, proliferation and differentiation^[194]^. The role of several miRNAs has been implied in CRC evolution and progression, moreover different miRNAs have been identified as predictive of treatment response to standard chemotherapy (i.e. miR-429 and miR-148a with 5-FU) and targeted agents (i.e. miR-7 and miR-375 with anti-EGFRs)^[195]^. Although promising these findings still need validation; nevertheless, the possible clinical application of miRNAs as biomarkers or as a potential target of treatment in CRC deserves further investigation. Of note, new strategies are currently under study to develop miRNA based inference methods to extensively infer drug-disease causal relationship (miRDDCR) to assist in experimental design for drug discovery and disease treatment^[196]^.

## CONCLUSION

In the era of precision medicine, optimizing therapeutics and drugs combination for a narrow subset of patients based on patients’ and tumors genetic makeup is of paramount importance in order to improve outcomes and minimize unrequired toxicities. The field of pharmacogenomics is constantly growing, and with the availability of new technologies it has been moving beyond candidate gene approaches and genome-wide association studies towards a comprehensive evaluation of genomic and epigenomic markers to drive treatment choices and optimize targeted therapies. Several biomarkers have entered clinical practice so far, and many more are currently being tested in clinical trials. Biomarker discovery and validation however still encounter many issues, due often to the small subsets of patients bearing selected alterations, the retrospective nature of most studies and the difficulty in proving the cost-effectiveness of a specific novel marker. Implementing biomarker-driven clinical trials and prospective pharmacogenomic profiling in clinical research, possibly integrating companion diagnostic tests since the early stages of novel drug development, is thus a priority for future research. Finally, dynamic profiling of tumor genomics under treatment pressure will play a critical role in uncovering acquired mechanism of resistance and directing personalized treatment strategies.

## Figures and Tables

**Table 1.** Summary of main presented biomarkers

Biomarker	Type of alteration	Frequency in CRC	Approved for clinical practice	Predictive value	Ref.
KRAS	Exon 2 (codons 12 and 13), exon 3 (codons 59 and 61) and exon 4 (codons 117 and 146) mutations	40%–50% mCRC	Y	Resistance to anti-EGFRs	[5]
NRAS	Exon 2 (codons 12 and 13), exon 3 (codons 59 and 61) and exon 4 (codons 117 and 146) mutations	3%–5% mCRC	Y	Resistance to anti-EGFRs	[5]
BRAF	V600E mutations	8%–10%	Y (prognostic value, Lynch Sdr screening in MSI-H)	Resistance to anti-EGFRs (accumulating evidence)	[5]
MSI	MMR-D (MSI-H)	20% stage I-II, 12% stage III, 4%–5% stage IV	Y (Lynch Sdr screening, prognostic value in early stage CRC)	Response to immune-checkpoint inhibitors (mCRC) Lack of efficacy of 5-FU adjuvant therapy in stage II (low evidence)	[5,81,100,101]
DPYD	DPYD*2A (IVS14+1G>A)	1%–2% heterozygous (caucasian population)	Y	5-FU severe toxicity	[9,120]
UGT1A1	UGT1A1*28	45% heterozygous 10% homozygous (caucasian population)	Y	Irinotecan severe toxicity	[9,10]
HER2	HER2 amplification	5% RAS WT mCRC	N	Resistance to anti-EGFRs Response to anti-HER2 treatment	[133–135]
PI3K	Exon 9 and 20 hotspot mutations	10%–18%	N	Resistance to anti-EGFRs	[5]
CIMP	Aberrant DNA hypermethylation at select CpG islands	10%–15%	N	Response to 5-FU adjuvant therapy Potential resistance to anti-EGFRs Potential sensitivity to demethylating agents	[161]
MGMT	MGMT promoter hypermethylation	40% mCRC	N	Response to alkylating agents	[172]

Y: yes; N: no; CRC: colorectal cancer; mCRC: metastatic CRC; EGFR: epidermal growth factor receptor; 5-FU: 5-fluorouracil; MSI-H: high microsatellite instability

**Table 2.** Promising future pharmacogenomics biomarkers

Biomarker	Description	Potential predictive value	Ref.
CMS1	Microsatellite instability immune (14%): - high TML -MSI -CIMP+ -BRAF mutation -strong immune activation -right sided	Response to anti-VEGF	[181–186]
CMS2	Canonical (37%): -epithelial signature -WNT-β-catenin and MYC activation -CIN -left sided	Response to anti-EGFRs Response to anti-HER2 Chemo-sensitivity	[181–186]
CMS3	Metabolic (13%): -metabolic dysregulation	-	[181–186]
CMS4	Mesenchymal (23%): -TGF-β activation -stromal invasion -angiogenesis	Resistance to anti-EGFRs Lack of benefit from 5-FU and oxaliplatin	[181–186]
Liquid biopsy	Mutational analysis of circulating tumor DNA	Identification of predictive mutations for targeted treatments at baseline Dynamic monitoring Early detection of secondary resistance	[187–191]
MiRNA	Micro RNA: noncoding single-stranded RNA molecules, < 200 nucleotides, with post-transcriptional regulatory functions	Response/resistance to chemotherapy and targeted agents	[195]

TML: tumor mutational load; EGFR: epidermal growth factor receptor; 5-FU: 5-fluorouracil; MSI: microsatellite instability; TGF: transforming growth factor; VEGF: vascular endothelial growth factor
